# The impact of tourniquet on tibial bone cement penetration in different zones in primary total knee arthroplasty: a meta-analysis

**DOI:** 10.1186/s13018-021-02345-1

**Published:** 2021-03-17

**Authors:** Changjiao Sun, Xin Yang, Xiaofei Zhang, Qi Ma, Peng Yu, Xu Cai, Yonggang Zhou

**Affiliations:** 1 Department of Orthopedic, Beijing Tsinghua Changgung Hospital, School of Clinical Medicine, Tsinghua University, No. 168 Litang Road, Dongxiaokou Town, Changping District, Beijing, 102218 China; 2 Department of Orthopedic, Peking University First Hospital, No. 8 Xishiku Street, Xicheng District, Beijing, China; 3 Department of Clinical Epidemiology and Biostatistics, Beijing Tsinghua Changgung Hospital, School of Clinical Medicine, Tsinghua University, No. 168 Litang Road, Dongxiaokou Town, Changping District, Beijing, China; 4 Department of Orthopedic, Wuhan University of Science and Technology Hospital, Qingling Street, Hongshan District, Wuhan, China; 5 Department of Orthopaedic Surgery, The First Medical Centre, Chinese PLA General Hospital, No. 28 Fuxing Road, Beijing, 100853 China

**Keywords:** Tourniquet, Total knee arthroplasty, Cement mantle, Penetration

## Abstract

**Background:**

Cement mantle penetration and the cement–bone interface strength were critical to a successful primary total knee arthroplasty (TKA). It remained unclear whether decreased blood and fat in the cancellous bone achieved with the use of a tourniquet increases tibial cement mantle penetration in different zones on AP and lateral view in TKA according to criteria defined by the Knee Society Scoring System (KSS). The purpose of this study was to determine whether tourniquet use influences tibial cement mantle penetration in different zones on AP and lateral view in TKA according to KSS.

**Methods:**

We conducted a meta-analysis to identify studies involving the impact of tourniquet use and no tourniquet use on tibial bone cement penetration in primary TKA in electronic databases, including Web of Science, Embase, PubMed, Cochrane Controlled Trials Register, Cochrane Library, Highwire, CBM, VIP, Wanfang database, up to January 2021. Finally, we identified 1231 patients (1231 knees) assessed in twelve studies.

**Results:**

Tourniquet use increases the cumulative cement mantle penetration (*P* < 0.00001), mean cement mantle penetration (*P* = 0.004), and cement mantle in zone 3(*P* < 0.0001) on AP view. However, there were no significant differences in cement mantle in zone 1(*P* = 0.5), zone 2(*P* =0 .54), zone 4(*P* = 0.07) on AP view, and zone 1(*P* = 0.32), zone 2(*P* = 0.38) on lateral view between two groups. There were also no significant differences in length of surgery(*P* = 0.7), change in hemoglobin(*P* = 0.4), transfusion rates(*P* = 0.47), and complications such as muscular calf vein thrombosis(*P* = 0.21), superficial infection (*P* = 0.72), and deep vein thrombosis (*P* = 0.66) between two groups.

**Conclusion:**

The application of a tourniquet increases the thickness of the tibial bone cement penetration—the increase in the thickness of bone cement penetration mainly located in zone 3 on the anteroposterior (AP) view.

## Background

Cement mantle penetration and the strength of the cement–bone interface are critical to a successful primary TKA. Because bone cement has no adhesive properties, adequate penetration is vital to achieve component stability by mechanical interlock with bony trabecular spaces [1–4]. Increased cement mantle thickness has also been shown to confer increased implant survival and stability [1, 5, 6]. One of the factors that can easily affect the cementation during TKA is a tourniquet during surgery [7]. The potential benefit of tourniquet use was to aid in preparing the bone surface for cementation by reducing the blood and fat in the field and offers better visualization due to bloodless field, which would facilitate cementing quality [8]. Some literature found tourniquet use will increase the bone cement penetration [7, 9–12]. However, some studies suggest that tourniquet use does not affect cement mantle penetration [13–17]. There is no consensus and evidence-based medicine of tourniquet use on bone cement mantle penetration, especially bone cement mantle penetration in a different zone. Therefore, in this meta-analysis, our specific purpose was to determine whether tourniquet use influences tibial cement mantle penetration in different zones on AP and lateral view in TKA based on the Knee Society scoring system (KSS) [18].

## Methods

According to the Preferred Reporting Items for Systematic Reviews and Meta-Analyses statement, we strictly followed the PRISMA (preferred reporting items for systematic review and meta-analysis) guidelines to conduct this analysis [19].

### Search strategy

We conducted a meta-analysis to identify studies involving the impact of tourniquet use and no tourniquet use on tibial bone cement penetration in primary TKA in electronic databases, including PubMed, Web of Science, Embase, Cochrane, Controlled Trials Register, Cochrane Library, Highwire, CBM, VIP, CNKI, Wanfang database, up to January 2021. The keywords used were “total knee arthroplasty,” “total knee replacement,” “tourniquet,” “bone cement mantle,” penetration,” in conjunction with Boolean operators “AND” or “OR.” We used the Review Manager Software was to perform the meta-analysis.

### Inclusion criteria

This review includes randomized controlled trials (RCTs) and non-randomized studies of interventions (NRSI) with a control group that comparing the impact of tourniquet use and no tourniquet use on tibial bone cement penetration in primary TKA. The included studies should meet the following inclusion criteria: (1) The TKA procedure was performed for the first time. (2) The impact of tourniquet use on tibial bone cement penetration was involved. (3) The comparator was the impact of no tourniquet use on tibial bone cement penetration in the original comparative study. (4) At least one of the following indexes was reported: the thickness of cement mantle penetration on the tibia, surgery duration, change in hemoglobin, transfusion rates, and complications such as muscular calf vein thrombosis (MCVT), superficial infection, and deep vein thrombosis (DVT). We also excluded (1) studies of TKA revision and (2) unclear or incomplete sample data were available.

### Data extraction process

**Fig. 1:**
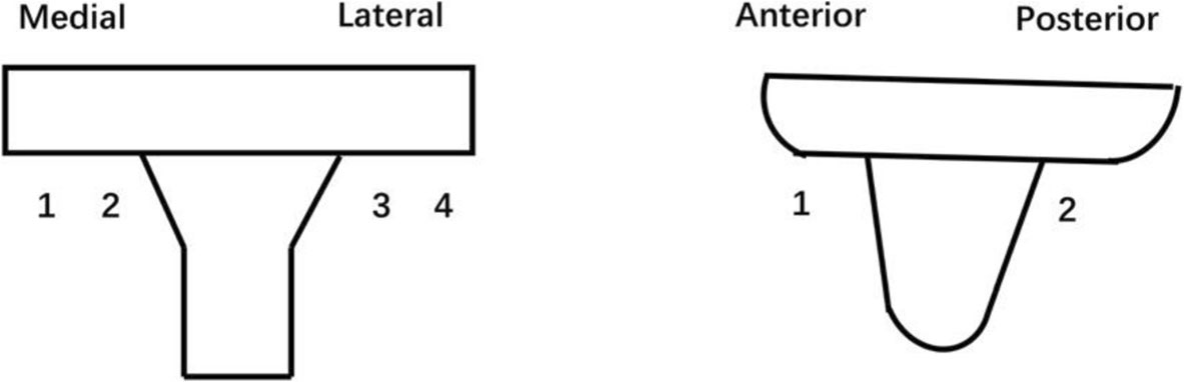
Tibial cement mantle in different zones on AP and lateral view according to KSS. Tibial cement mantle penetration in different zones on AP and lateral view in TKA according to KSS

All RCTs and NORIs comparing the impact of tourniquet use and no tourniquet use on tibial bone cement penetration with primary TKA were identified and included from the search strategy. Two researchers independently reviewed titles and abstracts to assess study eligibility against the predefined criteria and independently extracted the available data from each study. Disagreements on inclusion of studies and data extractions were discussed and a consensus reached. Data were extracted based on the following: (1) research features (i.e., authors, type of study and year of publication), (2) population information (i.e., gender, body mass index (BMI), (3) intervention (i.e., tourniquet pressure, tourniquet time, the brand of bone cement, cementing technique, drainage). The primary outcome measure was the thickness of cement mantle penetration on the tibia, including different zones on AP and lateral view in TKA based on the Knee Society scoring system(KSS). Tibial anteroposterior (AP) zones 1, 2, 3, and 4 (Fig. 1) represent the medial and lateral inferior surfaces of the tibial baseplate, respectively. Tibial lateral zones 1 and 2 (Fig. 1) represent the anterior and posterior distal surfaces of the tibial baseplate, respectively. For zones in both the AP and lateral tibial views, cement penetration was measured at the one-third, two-third, or one-half marks. The cumulative cement penetration depth was calculated and expressed as the sum of all measurement. Cement depth was measured utilizing the measurement tool in the picture archiving and communication system (PACS). Secondary outcomes were surgery time duration, change in hemoglobin, transfusion rates, and complications such as MCVT, superficial infection, and DVT.

### Assessment of studies

The two authors (C.J.S and X.Y.) independently assessed the risk of bias and quality of studies using the Cochrane Risk of Bias tool for randomized studies and the nine-star Newcastle-Ottawa scale (NOS) for NRSIs [20, 21]. Two researchers independently evaluated the studies, and disagreements were resolved through discussions with a third author or consensus. For this review, studies scoring ≤ 4 stars or at high risk of bias on Cochrane Risk of Bias tool were defined as being of low quality.

### Statistical analysis

A random-effects model was used due to expected methodological heterogeneity among studies in relation to differences in cementing technique, tourniquet pressure, tourniquet time, and bone cement brand. In each study, we commonly used the odds ratio (OR) and relevant 95% confidence interval (CI) to measure dichotomous variables such as rates of transfusion and complications such as muscular calf vein thrombosis, superficial infection, and deep vein thrombosis. Given that the outcome is rare, OR was supposed to approximate RR (relative risk) based on Cornfield's rare disease outcome assumption [22]. The mean difference (MD) was used to assess continuous outcomes such as cement mantle penetration, length of surgery, and hemoglobin change with a 95% confidence interval (CI). Statistical algorithms were used to estimate the standard deviation for those studies that provided only continuous variables for means and range [23]. Meta-analysis was undertaken using Review Manager (version 5.3 for MAC, the Cochrane Collaboration, the Nordic Cochrane Centre, and Copenhagen, 2014). We considered the results as a statistically significant difference if *p* values were less than 0.05.

## Results

### Search results

**Fig. 2:**
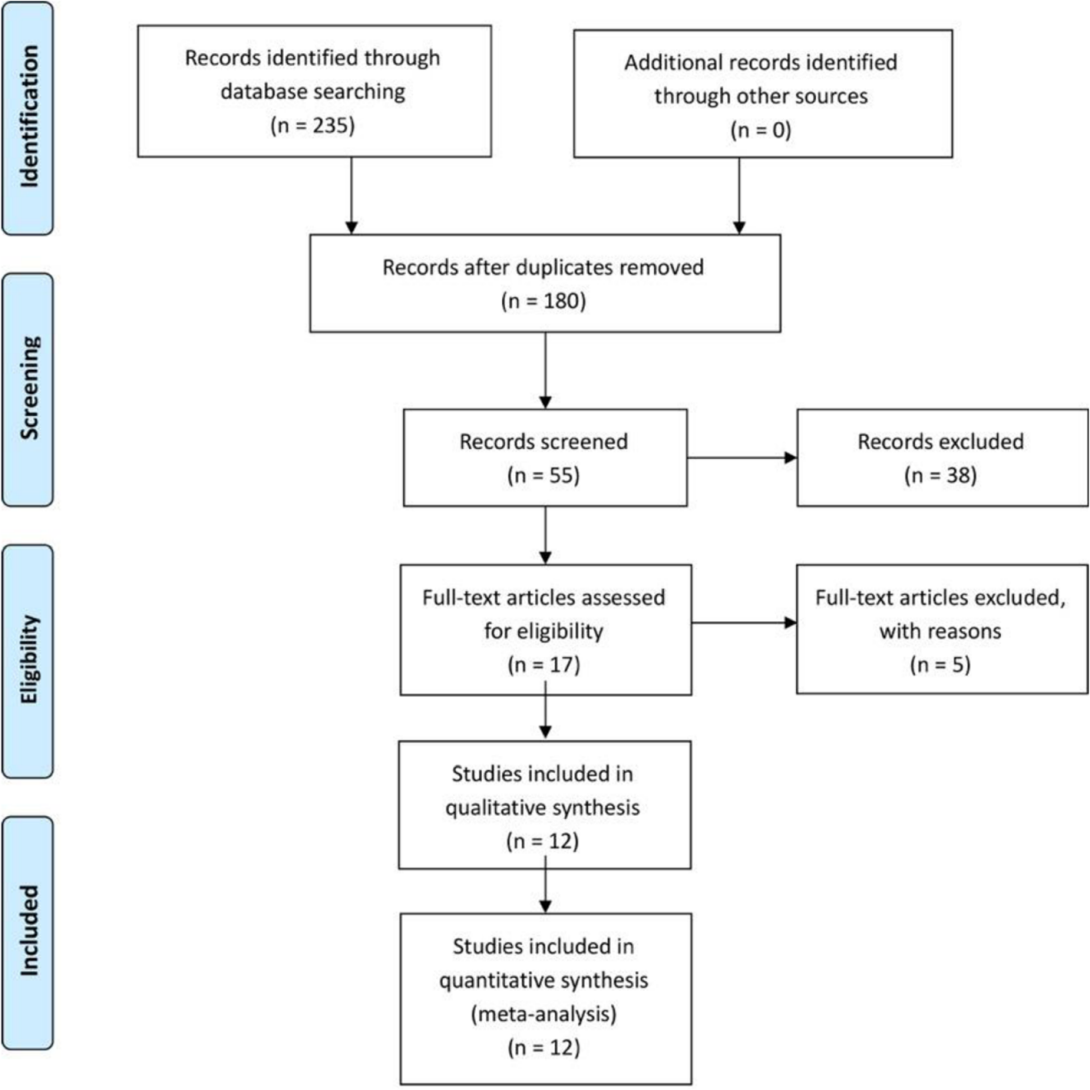
The search results and selection procedure. Two hundred thirty-five relevant citations were identified from the databases according to the literature search strategy described earlier. After deleting 180 duplicates, we obtained 55 studies. Based on screening titles and abstracts of the 55 remaining articles, 38 irrelevant clinical studies were excluded. By reading the 17 full-text articles, we excluded another 5 articles. Finally, we identified 1231 patients assessed in 12 articles

The literature search strategy and selection process are shown in Fig. 2. Finally, eleven publications from 2014 to 2021 were included in our meta-analysis. Two hundred thirty-five relevant citations were identified from the databases according to the literature search strategy described earlier. After deleting 180 duplicates, we obtained 55 studies. Based on screening titles and abstracts of the 55 remaining articles, 38 irrelevant clinical studies were excluded. By reading the 17 full-text articles, we excluded another 5 articles for the following reasons: none-compare groups and no useful outcome data. The remaining 12 articles were deemed appropriate. Finally, we identified 1231 patients (1231 knees) assessed in (8 RCTs [7, 10, 13–15, 17, 22, 24] and 4 NRSIs [9, 11, 12, 16])

### Study characteristics and quality

The characteristics of the 12 included studies are given in Tables 1 and 2. All included articles were published in English and Chinese between the years 2014 and 2021.

**Table 1:** Summary of studies characteristics

The detailed baseline characteristics information							
Tourniquet use /no tourniquet use							
Author/year	Patients	Knees	Mean age (years)	Female gender (%)	BMI	Diagnosis	Study type
**Gao 2019** [9]	29/29	29/29	62.3/63.7	75.9/69	25.72/26.13	29OA/29OA	Retrospective study
**Hegde 2021** [12]	61/61	61/61	63.64/63.66	34.3/34.3	30.43/29.48	NA	Retrospective study
**Herndon 2020** [16]	70/70	70/70	67/67.5	40/37.1	NA	NA	Retrospective study
**Jawhar 2019** [13]	43/43	43/43	70/71	62.8/62.8	31.9/31.9	43OA/43OA	RCT
**Ozkunt 2018** [14]	24/25	24/25	NA	NA	NA	24OA/25OA	RCT
**Pfitzner 2016** [7]	45/45	45/45	69.3/70.5	53.3/75.6	27.8/26	45OA/45OA	RCT
**Touzopoulos 2019** [10]	50/50	50/50	70.73/69.92	16/16	31.04/31.32	50OA/50OA	RCT
**Vertullo 2017** [15]	20/20	20/20	67.85/65.65	50/45	30.43/31	20OA/19OA+1RA	RCT
**Xie 2017** [17]	45/45	45/45	66.2/66.1	85/75	26.1/25.9	NA	
**Yang 2017** [24]	41/41	41/41	62.8/66.3	80.5/83	NA	41OA/41OA	RCT
**Gapinski 2019** [11]	91/79	91/79	67.4/66.9	67/67	33.6/35	NA	Retrospective study
**Zhou 2018** [22]	49/49	49/49	62.7/62.5	47/51	26.1/26.4	49OA/49OA	RCT

The detailed baseline characteristics information including the number of TKAs, age, gender, BMI, diagnosis, and type of studies of two groups

*Abbreviations*: *OA* osteoarthritis, *RA* rheumatoid arthritis, *BMI* body mass index, *RCT* randomized controlled trial

**Table 2:** The detailed characteristics of general intervention information

Author/year	Tourniquet pressure	Tourniquet time	TXA	Bone cement	Cementing technique	Mantle measurement	Drainage
**Gao 2019** [9]	280-350 mmHg	From incision until final closure	No	NS	Third-generation	Cumulative penetration depth	No
**Hegde 2021** [12]	250 mmHg	From incision until cementation	No	Simplex P	Fourth-generation	Cumulative penetration depth, AP zone 1,4,Lateral zone 12	NS
**Herndon 2020** [16]	250 mmHg	From incision until final closure	Yes	Simplex	Third-generation	Cumulative penetration depth	NS
**Jawhar 2019** [13]	360 mmHg	From incision until final closure	No	SmartSet	Third-generation	Cumulative penetration depth, AP zone 1,4,Lateral zone 12	Yes
**Ozkunt 2018** [14]	NS	From incision until final closure	No	OrCem 3	Third-generation	AP zone 1,4,Lateral zone 12	NS
**Pfitzner 2016** [7]	350 mmHg	From incision until final closure	No	Palacos R	Fourth-generation	Cumulative bone cement	Yes
**Touzopoulos 2019** [10]	350 mmHg	From incision until final closure	Yes	Palacos R+G	Fourth-generation	AP zone 1,4,Lateral zone 12	Yes
**Vertullo 2017** [15]	300 mmHg	From incision until final closure	No	Palacos R+G	Third-generation	Average penetration depth	NS
**Xie 2017** [17]	100 mmHg above systolic pressure	From incision until final closure	Yes	NS	Third-generation	Average penetration depth	Yes
**Yang 2017** [24]	100 mmHg above systolic pressure	From incision until final closure	No	SmartSet	Third-generation	Cumulative penetration depth	Yes
**Gapinski 2019** [11]	NS	From incision until final closure	Yes	NS	Third-generation+CO2 gas	AP zone 1,4,Lateral zone 12	NS
**Zhou 2018** [22]	100 mmHg above systolic pressure	From incision until final closure	Yes	NS	Third-generation	Average penetration depth	Yes

The detailed information of tourniquet pressure, tourniquet realizing time, TXA administration, and thromboprophylaxis of two groups

### Risk of bias assessment

**Fig. 3:**
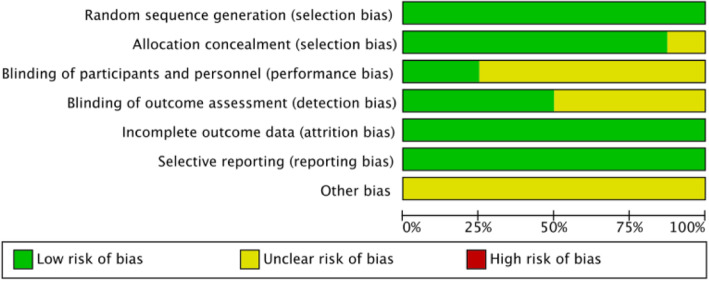
Risk of bias graph. Review authors’ judgements about each risk of bias item presented as percentages across all included studies

**Fig. 4:**
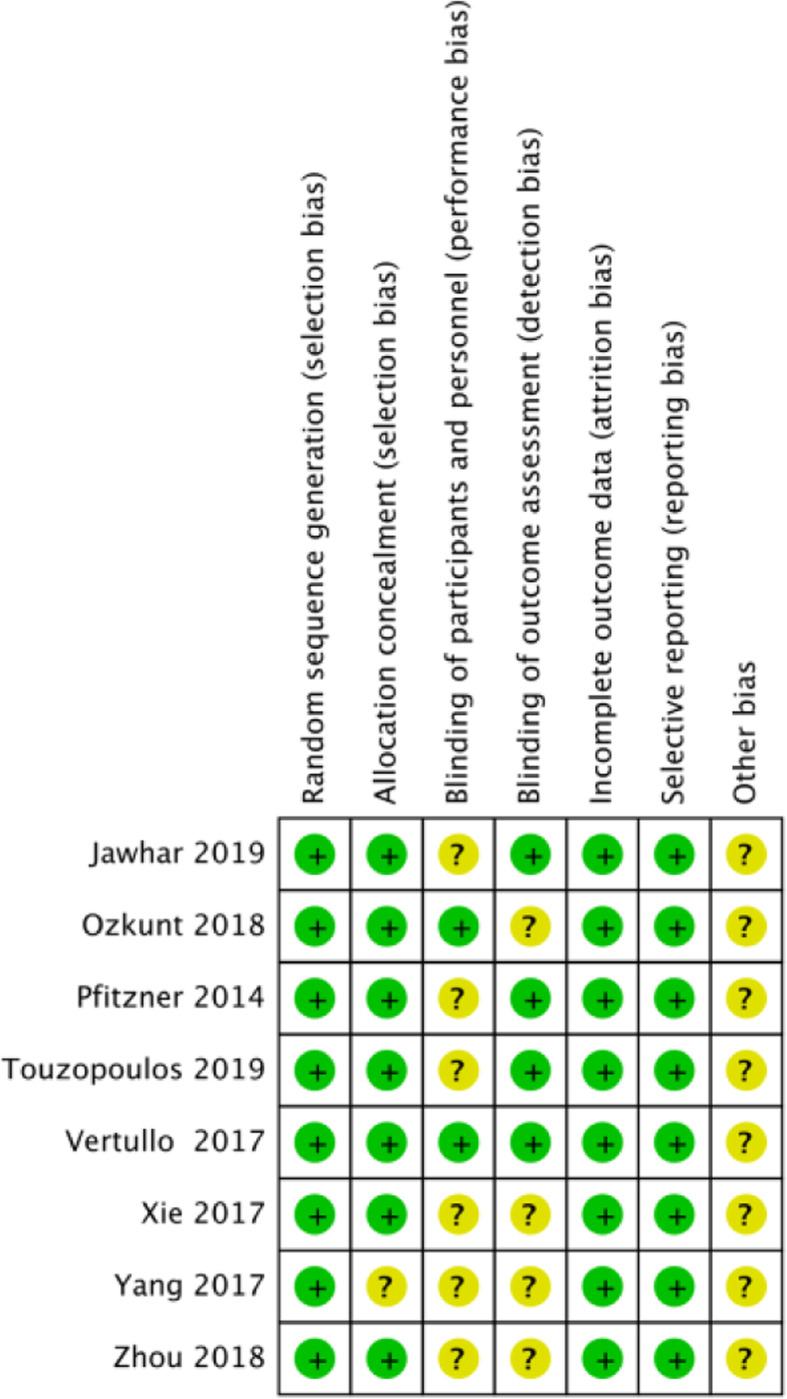
Risk of bias summary. Review authors’ judgements about each risk of bias item for each included study

The methodological quality of the involved NRSIs ranged from seven to eight (Table 3). The risk of bias summary and risk of bias graph for RCTs are shown in Figs. 3 and 4. As a result, the overall quality of all included studies was considered adequate.

**Table 3:** Risk-of-bias assessment for the studies included in the meta-analysis (NOS)

Risk-of-bias assessment for the studies included in the meta-analysis (NOS)									
(nRCT) Study = 6	Selection				Comparability	Outcome/exposure			Score
	Item 1	Item 2	Item 3	Item 4	Item 5	Item 6	Item 7	Item 8	
**Gao 2019** [9]	*	*		*	*	*	*	*	7
**Hegde 2021** [12]	*	*		*	**	*	*	*	8
**Herndon 2020** [16]	*	*		*	**	*	*	*	8
**Gapinski 2019** [11]	*	*		*	**	*	*	*	8

The methodological quality of the involved studies ranged from seven to eight

### Cumulative thickness of cumulative cement mantle penetration

**Fig. 5:**
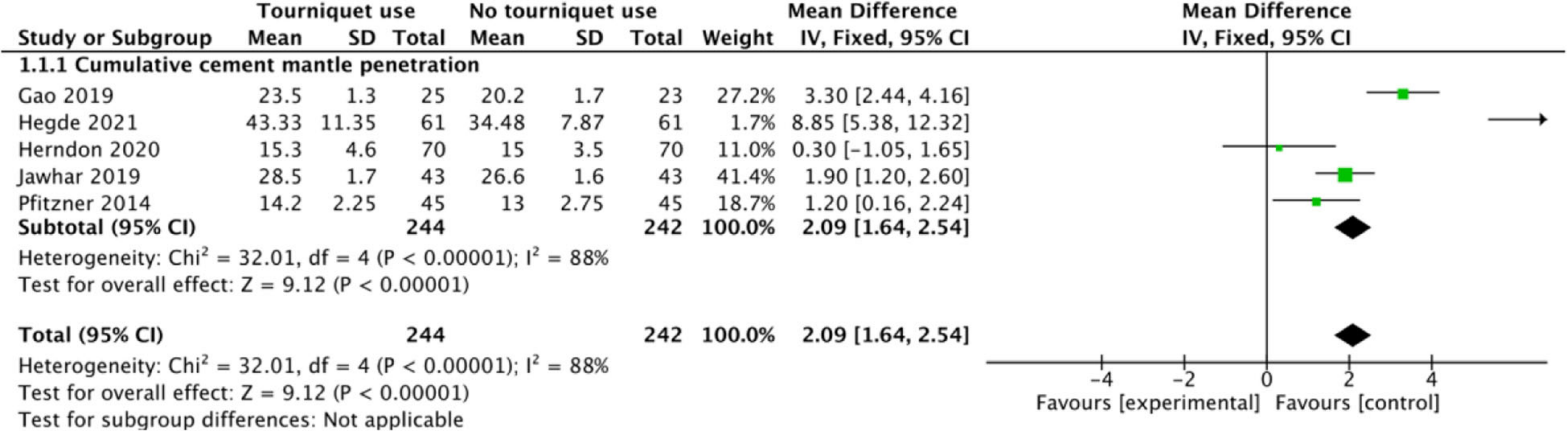
A forest plot diagram showing cumulative cement mantle penetration. Five studies assessed the thickness of cumulative cement mantle penetration. The meta-analysis results showed a significant difference in cumulative thickness of cumulative cement mantle penetration between the tourniquet use group and no tourniquet use group (MD 2.09, 95% CI 1.64 to 2.54, *P* < 0.00001, *I*^2^ = 88%)

Five studies assessed the thickness of cumulative cement mantle penetration. The meta-analysis results showed a significant difference in cumulative thickness of cumulative cement mantle penetration between the tourniquet use group and no tourniquet use group (MD 2.09, 95% CI 1.64 to 2.54, *P* < 0.00001, *I*^2^ = 88%, Fig. 5). The results indicated that tourniquet use could increase the cumulative thickness of cement mantle penetration compared with the no tourniquet use group.

### Mean thickness of cement mantle penetration

**Fig. 6:**
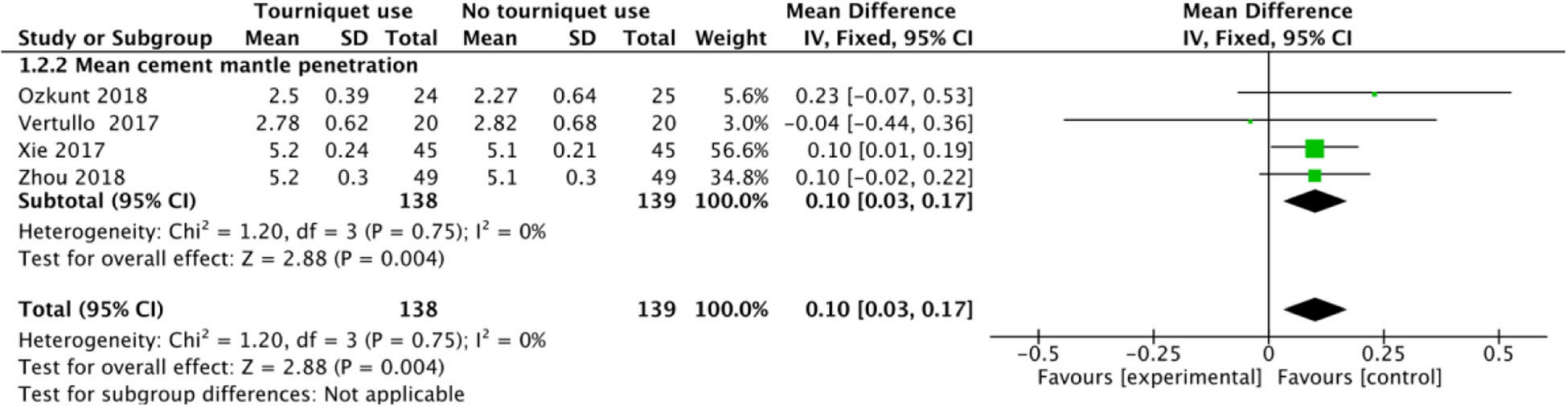
A forest plot diagram showing mean cement mantle penetration. Four studies reported the mean cement mantle penetration. The results showed significant difference in mean thickness of cement mantle penetration between the 2 groups (MD 0.1, 95% CI 0.03 to 0.17, *P* = 0.004, *I*^2^ = 0%)

Four studies reported the mean cement mantle penetration. The results showed significant difference in mean thickness of cement mantle penetration between the 2 groups (MD 0.1, 95% CI 0.03 to 0.17, *P* = 0.004, *I*^2^ = 0%; Fig. 6). The results indicated that compared with the no tourniquet use group, the tourniquet use could increase the mean thickness of cement mantle penetration.

### The thickness of cement mantle penetration in different zones on AP and lateral view

**Fig. 7:**
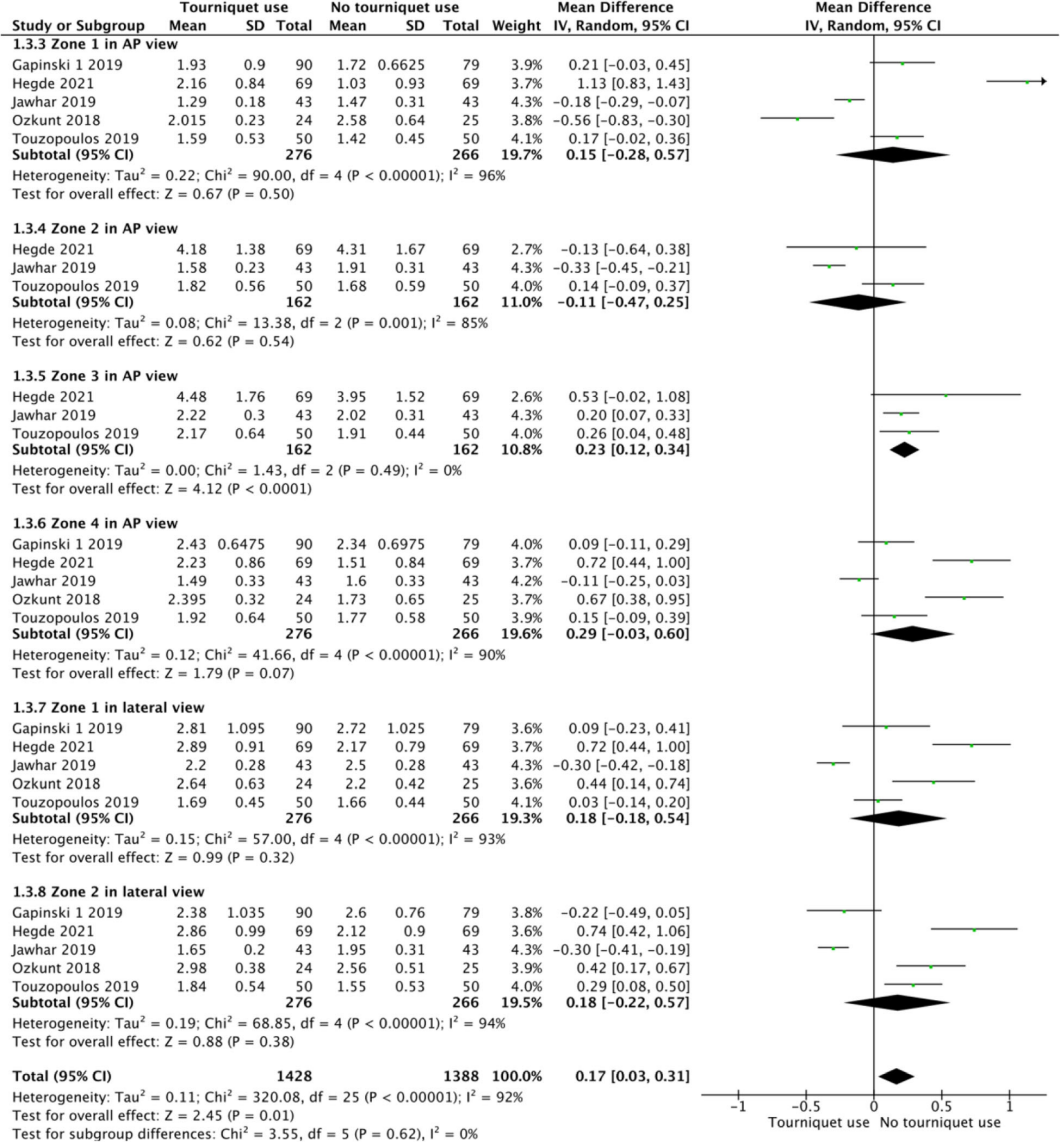
A forest plot diagram showing cement mantle penetration in different zones on AP and lateral view. Five studies reported the cement mantle penetration in zone 1 on AP view. The results showed no significant difference in cement mantle penetration in zone 1 on AP view between the 2 groups (MD 0.15, 95% CI - 0.28 to 0.57, *P* = 0.5, *I*^2^ = 96%). Three studies reported the cement mantle penetration in zone 2 on AP view. The results showed no significant difference in cement mantle penetration in zone 2 on AP view between the 2 groups (MD - 0.11, 95% CI - 0.47 to 0.25, *P* = 0.54, *I*^2^ = 85%). Three studies reported the cement mantle penetration in zone 3 on AP view. The results showed significant difference in thickness of cement mantle penetration in zone 3 on AP view between the 2 groups (MD 0.23, 95% CI 0.12 to 0.34, *P* < 0.0001, *I*^2^ = 0%). Five studies reported the cement mantle penetration in zone 4 on AP view. The results showed no significant difference in cement mantle penetration in zone 4 on AP view between the 2 groups (MD 0.29, 95% CI - 0.03 to 0.6, *P* = 0.07, *I*^2^ = 90%). Five studies reported the cement mantle penetration in zone 1 on lateral view. The results showed no significant difference in cement mantle penetration in zone 1 on lateral view between the 2 groups (MD 0.18, 95% CI - 0.18 to 0.54, *P* = 0.32, *I*^2^ = 93%). Five studies reported the cement mantle penetration in zone 2 on lateral view. The results showed no significant difference in cement mantle penetration in zone 2 on lateral view between the 2 groups (MD 0.18, 95% CI - 0.22 to 0.57, *P* = 0.38, *I*^2^ = 94%)

Five studies reported the cement mantle penetration in zone 1 on AP view. The results showed no significant difference in cement mantle penetration in zone 1 on AP view between the 2 groups (MD 0.15, 95% CI - 0.28 to 0.57, *P* = 0.5, *I*^2^ = 96%, Fig. 7). Three studies reported the cement mantle penetration in zone 2 on AP view. The results showed no significant difference in Cement mantle penetration in zone 2 on AP view between the 2 groups (MD - 0.11, 95% CI - 0.47 to 0.25, *P* = 0.54, *I*^2^ = 85%, Fig. 7). Three studies reported the cement mantle penetration in zone 3 on AP view. The results showed significant difference in thickness of cement mantle penetration in zone 3 on AP view between the 2 groups (MD 0.23, 95% CI 0.12 to 0.34, *P* < 0.0001, *I*^2^ = 0%, Fig. 7). Five studies reported the cement mantle penetration in zone 4 on AP view. The results showed no significant difference in cement mantle penetration in zone 4 on AP view between the 2 groups (MD 0.29, 95% CI - 0.03 to 0.6, *P* = 0.07, *I*^2^ = 90%, Fig. 7). Five studies reported the cement mantle penetration in zone 1 on lateral view. The results showed no significant difference in cement mantle penetration in zone 1 on lateral view between the 2 groups (MD 0.18, 95% CI - 0.18 to 0.54, *P* = 0.32, *I*^2^ = 93%, Fig. 7). Five studies reported the cement mantle penetration in zone 2 on lateral view. The results showed no significant difference in cement mantle penetration in zone 2 on lateral view between the 2 groups (MD 0.18, 95% CI - 0.22 to 0.57, *P* = 0.38, *I*^2^ = 94%, Fig. 7).

### Duration of surgery time

**Fig. 8:**
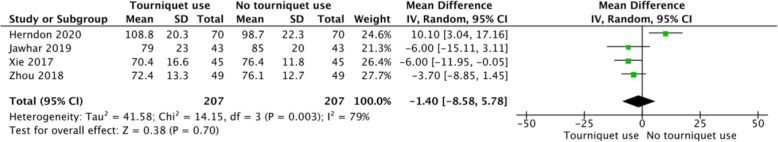
A forest plot diagram showing duration of surgery time. Four studies reported the length of surgery time. The results showed no significant difference in duration of surgery time between the 2 groups (MD - 1.4, 95% CI - 8.58 to 5.78, *P* = 0.70, *I*^2^ = 79%)

Four studies reported the length of surgery time. The results showed no significant difference in duration of surgery time between the two groups (MD - 1.4, 95% CI - 8.58 to 5.78, *P* = 0.70, *I*^2^ = 79%; Fig. 8)

### Change in hemoglobin

**Fig. 9:**
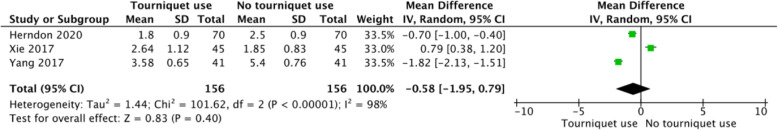
A forest plot diagram showing change in hemoglobin. Three studies reported the Change in hemoglobin. The results showed no significant difference in change in hemoglobin between the 2 groups (MD - 0.58, 95% CI - 1.95 to 0.79, *P* = 0.4, *I*^2^ = 98%)

Three studies reported the change in hemoglobin. The results showed no significant difference in change in hemoglobin between the two groups (MD - 0.58, 95% CI - 1.95 to 0.79, *P* = 0.4, *I*^2^ = 98%; Fig. 9)

### Blood transfusion rate

**Fig. 10:**
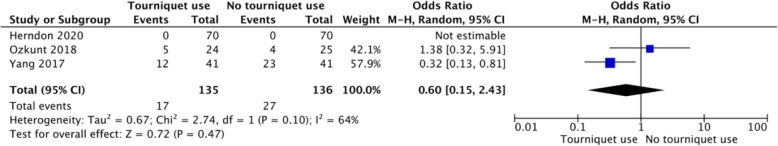
A forest plot diagram showing blood transfusion rate. Three studies reported the blood transfusion rate. The results showed no significant difference in blood transfusion rate between the 2 groups (OR 0.6, 95% CI 0.15 to 2.43, *P* = 0.47, *I*^2^ = 64%)

Three studies reported the blood transfusion rate. The results showed no significant difference in blood transfusion rate between the two groups (OR 0.6, 95% CI 0.15 to 2.43, *P* = 0.47, *I*^2^ = 64%; Fig. 10)

### Complications

**Fig. 11:**
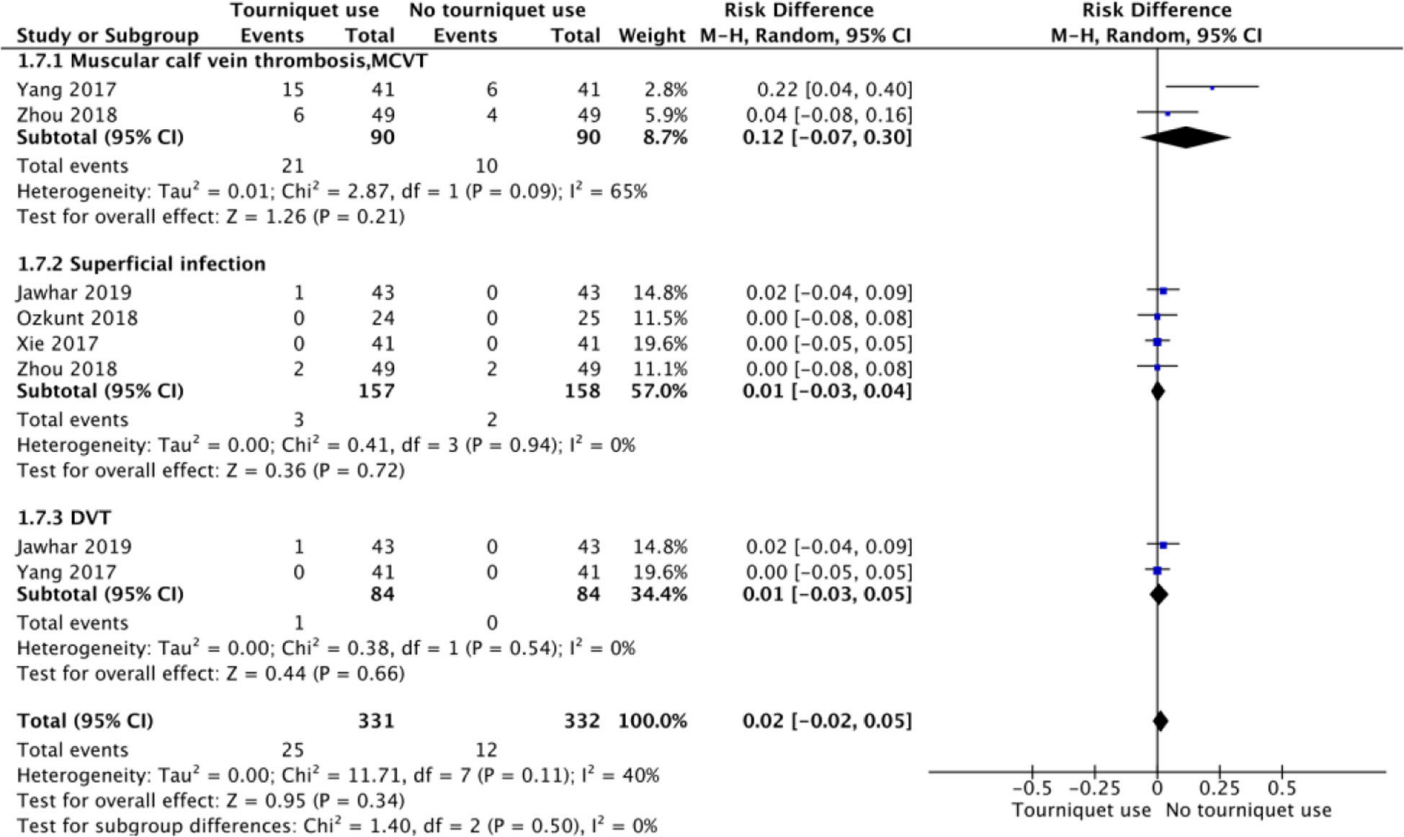
A forest plot diagram showing complications. Two studies reported the rate of MCVT. The results showed no significant difference in rate of MCVT between the 2 groups (OR 0.12, 95% CI - 0.07 to 0.3, *P* = 0.21, *I*^2^ = 65%). Two studies reported the rate of superficial infection. The results showed no significant difference in rate of superficial infection between the 2 groups (OR 0.01, 95% CI - 0.03 to 0.04, *P* = 0.71, *I*^2^ = 0%). Two studies reported the rate of DVT. The results showed no significant difference in rate of superficial infection between the 2 groups (OR 0.01, 95% CI - 0.03 to 0.05, *P* = 0.66, *I*^2^ = 0%)

Two studies reported the rate of MCVT. The results showed no significant difference in rate of MCVT between the 2 groups (OR 0.12, 95% CI - 0.07 to 0.3, *P* = 0.21, *I*^2^ = 65%; Fig. 11). Two studies reported the rate of superficial infection. The results showed no significant difference in the rate of superficial infection between the two groups (OR 0.01, 95% CI - 0.03 to 0.04, *P* = 0.71, *I*^2^ = 0%; Fig. 11). Two studies reported the rate of DVT. The results showed no significant difference in the rate of superficial infection between the two groups (OR 0.01, 95% CI - 0.03 to 0.05, *P* = 0.66, *I*^2^ = 0%; Fig. 11).

## Discussion

To the best of our knowledge, our study is the first meta-analysis to identify the tibial bone cement penetration in different zones on AP and lateral view according to the KSS with and without tourniquet application in primary TKA. There was only one meta-analysis identify the tibial bone cement penetration in TKA. However, they did not evaluate tibial cement mantle penetration in different zones on AP and lateral view. Our meta-analysis of 12 studied that evaluated a total of 1231 TKAs shows that the use of tourniquet increased the tibial cement mantle thickness in primary TKA. The increase of tibial cement mantle is mainly located in zone 3 on AP view. However, there no increase of tibial cement mantle in other zones, including zone 1, zone 2, zone 4 on AP view, and zone 1, zone 2 on the lateral view. There were also no significant differences in surgery time duration, change in hemoglobin, transfusion rates, and complications such as MCVT, superficial infection, and DVT between the two groups.

It is well known that increased initial fixation strength of the tibial component is an essential factor influencing the implant's continued function. Aseptic loosening is a devastating complication that usually occurs at the bone–cement interface [23]. Therefore, Increased cement penetration thickness is of paramount importance in creating an ideal cement–bone bond, determining the strength of the implant against shearing forces [6, 25, 26], and has also been shown to confer improved implant stability and survival [1, 5, 6]. Several studies [7, 13, 27, 28] have suggested that the optimal thickness of cement penetration is 3 to 4 mm for maximal cement–bone interface fixation. There are many factors that will improve the cement penetration such as uniform bone density with sufficient drill-hole interdigitation, reduced intraoperative bleeding [29–31], pulsed lavage [32–35], absence of sclerosis [33, 36], bone debris in cancellous bone [35, 37, 38], and blood at the cement–bone interface [32]. Some studies have found that tourniquet use leads to increased cement penetration [7, 39]. One potential advantage of using a tourniquet in cemented TKR is increased bone cement penetration due to decreased cancellous bone bleeding or clot debris during cementing [7]. Another advantage is that the tourniquet application offers better visualization due to the bloodless field, which would facilitate cementing quality [8]. Using a tourniquet can significantly decrease intraoperative blood loss and operation time but do not significantly decrease the rate of transfusion or DVT in TKA [40].

The result showing that the use of a tourniquet increases the tibial cement mantle thickness was consistent with the outcome of a previous meta-analysis [41]. However, in the previous study, they did not evaluate tibial cement mantle penetration in different zones on AP and lateral view. In our research, we found the increase of tibial cement mantle mainly located in zone 3 on AP view, and there was no increase of tibial cement mantle in other zones including zone 1, zone 2, zone 4 on AP view and zone 1, zone 2 on the lateral view. An explanation for the increase of the tibial cement mantle mainly located in zone 3 on AP view may be that zone 3 of the tibial cement mantle happens to be the most stressed area when the prosthesis is placed.

Hemoglobin change level and transfusion rate have been recognized as the most objective indicators of actual blood loss. In our study, there are no differences in hemoglobin change level and transfusion rate between the two groups, which was consistent with the outcome of the previous meta-analysis [40]. Tourniquet use may decrease intraoperative blood loss. However, Tourniquet release can result in ongoing bleeding from cut cancellous bone [42], blood extravasated into the knee joint and adjacent soft tissues [43], or blood loss from hemolysis [44].

A tourniquet will provide surgeons with a bloodless surgery field to facilitate the clear identification of anatomical structures with less electrocoagulation and wound irrigation during surgery, which might help shorten the operation time. However, our result showed tourniquet use did not reduce the duration of surgery time in our meta-analysis.

One of the more significant clinical concerns regarding tourniquet use is its association with thromboembolism. No significant difference was found between groups regarding the rate of IMVT and DVT in our study, which was consistent with Jawhar’s meta-analysis [45]. However, the complication of venous thrombosis is the secondary outcome, the number of included studies comparing venous thrombosis between two groups is little, although several studies have investigated the incidence of venous thrombosis using the tourniquet [46–50]. The evidence is mixed because of heterogeneous study groups, designs, and confounding factors like time of surgery making it difficult to compare.

Although we evaluated the impact of tourniquet on tibial bone cement penetration in different zones, we should know it is also very important to have a proper distribution of the cement through the porosity of the bone. Too much thickness of the mantle may imply danger to the bone as it goes deeper and increases necrosis risk. It is true and known from all the authors that bloodless field is better for the cementing time, so tourniquet should be applied in the cementing technique to get a proper interlocking mechanism of this tool. Neither this mechanism nor the thickness of the mantle allows a good fixation of the tibial baseplate.

## Limitations

The current meta-analysis has several limitations: first, there is a high heterogeneity because of differences in cementing technique, tourniquet pressure, time of tourniquet use, and the brand of bone cement. This heterogeneity may influence the reliability of results. So, we used a random-effects model for all analyses. Second, the depth of cement mantle was assessed in the simple post-op X-ray; many factors could affect the outcomes and the analysis of the thickness of the mantle. Third, only the cement mantle of the tibial component was analyzed. It is difficult to assess the femoral component cement mantle thickness on a lateral fluoroscopic radiograph because of the medial and lateral condyles’ overlay. However, as both tibia and femur were prepared and cemented simultaneously in a similar fashion intraoperatively, analysis of the tibial component alone is sufficient. Third, there are no worldwide uniform guidelines for performing total knee arthroplasty. Different surgical techniques (such as the selection of approach, anesthesia methods, patellar resurfacing, and type of prosthesis) were used in the individual studies. Lastly, although it has been shown that increased cement mantle thickness improved implant stability, our study only evaluates cement mantle thickness and does not assess the association of the thickness with long-term TKA implant survivorship or longevity. Further studies assessing the association of this thickness with long-term outcomes are necessary.

## Data Availability

The datasets generated during and/or analyzed during the current study are available from the corresponding author on reasonable request.

## Abbreviations

CIs: Confidence intervals
RCTs: Randomized controlled trials
NRSIs: Non-randomized studies of interventions
RR: Risk ratio
OR: Odds ratio
VMD: Weighted mean difference
TKA: Total knee arthroplasty
BMI: Body mass index
MCVT: Muscular calf vein thrombosis
DVT: Deep vein thrombosis
